# Homocysteine exaggerates microglia activation and neuroinflammation through microglia localized STAT3 overactivation following ischemic stroke

**DOI:** 10.1186/s12974-017-0963-x

**Published:** 2017-09-18

**Authors:** Shuang Chen, Zhiping Dong, Man Cheng, Yaqian Zhao, Mengying Wang, Na Sai, Xuan Wang, Huan Liu, Guowei Huang, Xumei Zhang

**Affiliations:** Department of Nutrition and Food Science, School of Public Health, Tianjin Medical University, Tianjin, 300070 China

**Keywords:** Homocysteine, Microglial cell, Inflammation, Ischemic brain, Signal transducers and activators of transcription 3

## Abstract

**Background:**

Elevated plasma homocysteine (Hcy) levels have been indicated as a strong and modifiable risk factor of ischemic stroke; the previous studies have shown that exposure to Hcy activates cultured microglia. However, whether neurotoxicity of Hcy involves microglia activation following brain ischemia and the underlying mechanisms remains incompletely understood.

**Methods:**

The cerebral damage was evaluated by staining with 2,3,5-triphenyltetrazolium chloride, hematoxylin-eosin, and Fluoro Jade B. The activation state of microglia was assessed via immunoreaction using the microglial markers Iba1 and OX-42. Then, the inflammatory factors such as tumor necrosis factor α (TNF-α), interleukin 6 (IL-6), and phosphorylated signal transducer and activator of transcription 3 (pSTAT3) were examined by Western blot analysis and fluorescence immunohistochemistry.

**Results:**

Elevated Hcy level augmented brain damage and neural cell toxicity in the brain cortex and the dentate gyrus region of the hippocampus after cerebral ischemia/reperfusion. Meanwhile, Hcy activated microglia and induced the expression of the inflammatory factors such as TNF-α and IL-6. Moreover, Hcy caused an increase in pSTAT3 expression which occurs in microglial cells. AG490, a JAK2-STAT3 inhibitor, effectively inhibited the phosphorylation of STAT3, microglial cell activation and the secretion of IL-6, TNF-*α* raised by Hcy treatment.

**Conclusions:**

STAT3 signaling pathway located in microglia plays a critical role in mediating Hcy-induced activation of microglia and neuroinflammation in rat MCAO model. This suggests the feasibility of targeting the JAK2/STAT3 pathway as an effective therapeutic strategy to alleviate the progression of Hcy-associated ischemia stroke.

**Electronic supplementary material:**

The online version of this article (10.1186/s12974-017-0963-x) contains supplementary material, which is available to authorized users.

## Background

Ischemic stroke is one of the most frequent causes of injury to the central nervous system (CNS). Complex pathological mechanisms are involved in brain injury after cerebral ischemia. Inflammatory processes have a fundamental role in the pathophysiology of ischemic injury stroke. Microglial cells, which are rapidly and time-dependently activated after ischemia, are considered as the major cellular contributors to post-injury inflammation [1, 2]. Stroke-induced microglial activation causes release of a variety of inflammatory mediators many of which are cytotoxic and/or cytoprotective. Emerging evidence demonstrates that alternatively activated microglia promotes tissue repair by secreting anti-inflammatory mediators and growth factors [3, 4]. In contrast, overactivated microglia following ischemia stroke can secrete a wide range of inflammatory factors and neurotoxic compounds, including nitric oxide, tumor necrosis factor (TNF)-α, interleukin (IL)-6, and reactive oxygen species (ROS), which are deleterious to bystander neurons and impact their processes [5]. As a result of findings such as these, it is increasingly well-accepted that microglia activation has been known to exert dual effects on ischemic injury. An improved understanding of the mechanisms underlying microglial activation and their functional modulation by the local microenvironment is likely to advance our knowledge of many CNS pathological states.

Homocysteine (Hcy) is an intermediate sulfhydryl-containing amino acid derived from methionine. It has been proved that patients suffering with severe hyperhomocysteinemia manifest typical clinical cardiovascular symptoms as well as neurological disorders, such as cerebral atrophy, dementia, and seizures [6]. The mechanism underlying Hcy mediated-pathogenesis of nervous system disorders is not yet fully understood. The toxicity of Hcy to CNS neurons is widely recognized affecting both the neuronal survival rate and the ability of neurons to transmit signal and thus to form functional neural networks [7]. Moreover, Hcy is deemed to affect microglia proliferation in vitro [8]. However, whether microglia activation and microglia-mediated inflammatory responses involve neurotoxicity of Hcy remains largely unknown.

The signal transducer and activator of transcription 3 (STAT3), a member of the STAT protein family of transcription factors, has been extensively described as a central signaling molecule that controls cellular adaption in response to environmental stimuli or stress. Several groups have shown that the STAT3 is activated in vitro and in vivo experimental models of stroke and subsequently activated STAT3 promotes numerous genes responsible for many cellular functions that may play a critical role in both neural injury and repair [9, 10]. There are conflicting data whether this pathway activation leads to improved neurological recovery. Some findings suggest that treatments that activate the STAT3 signaling after experimental stroke may lead to improved functional performance and/or decreased cell death [11]. On the contrary, accumulating evidence suggests that activation of STAT3 leads to decreased cerebral recovery and that blocking this pathway leads to better neurological outcomes [12]. Thus, the precise contribution of STAT3 activation after stroke remains incompletely understood. On the other hand, STAT3, an important regulator of inflammatory gene expression, has been shown to play an important regulatory role in microglial reactivity to various stimuli and mediate pro-inflammatory responses in microglia in response to various CNS insults. Activated STAT3 can promote the transcription and expression of many genes that encode proinflammatory mediators, including cytokines, chemokines, adhesion molecules, and inflammatory enzymes [2]. In addition, it has been demonstrated that neuroinflammatory processes after cerebral ischemia involve aberrantly activated STAT3. The strong induction of pSTAT3 is predominantly localized in the macrophages/microglia in the post-ischemic brain [12]. Given the importance of STAT3 in activating microglia and inducing cytokine/chemokine expression, we investigated whether Hcy activates STA﻿T3 in microgla and whether STAT3 is involved in the Hcy-induced inflammatory responses.

Accordingly, the present study was designed to elucidate the molecular mechanism of neurotoxicity by Hcy in relation to microglial-mediated neuroinflammation and microglial STAT3 overactivation using a rat model of transient focal cerebral ischemia. This study demonstrates for the first time that STAT3 overactivation located in microglia cells plays an important role in Hcy-induced microglia activation and the inflammatory responses both in the brain cortex and the dentate gyrus (DG) region of the hippocampus following ischemic injury.

## Methods

### Experimental animals and administration

One hundred adult male Sprague-Dawley rats (180–220 g) (Grade SPF, Certificate Number SCXK (jing) 2012–0001) were purchased from Vital River Laboratory Co. Ltd. (Beijing, China). The experimental protocols were approved by the Tianjin Medical University Animal Ethics Committee and performed in compliance with institutional guidelines under approved protocols. The rats were randomly allocated into six groups: SHAM operation control group (SHAM), middle cerebral artery occlusion reperfusion group (MCAO), Hcy-treated SHAM group (SHAM + Hcy), Hcy-treated MCAO group (MCAO + Hcy), Hcy combined with AG490(α-cyano-(3,4-dihydroxy)-N-benzylcin-namide)-treated SHAM group (SHAM+ Hcy + AG490), and Hcy combined with AG490-treated MCAO group (MCAO + Hcy + AG490). Hcy (1.6 mg/kg/day, Sigma-Aldrich, St. Lous, Mo, USA) was administered by tail vein injection (Additional file 1: Video S1) for 21 days prior to SHAM or MCAO operation. The AG490 (3 mg/kg, Abcam, Cambridge, MA, USA) was administered by intraperitoneal injection before 1 h of SHAM or MCAO operation.

### Induction of focal cerebral ischemia-reperfusion

The MCAO rats were induced by using the intraluminal filament technique as we described previously [13]. A nylon thread was advanced by the left common carotid artery, through the left internal carotid artery and into the origin of the middle cerebral artery. Two hours after cerebral ischemia, the thread was carefully withdrawn to establish reperfusion. Animals assigned to SHAM operation were treated similarly, except that the thread was not advanced to the origin of the middle cerebral artery.

### Measurement of infarct volume

At 24 h after reperfusion, three rats in each group were euthanized and the brains were rapidly removed and frozen at −20 °C for 15 min. Serial coronal brain slices with a 2 mm thickness each were stained with 2% 2,3,5-triphenyltetrazolium chloride (TTC) at 37 °C for 15 min followed by fixation with 4% paraformaldehyde for 24 h. Infarct volume measurements were carried out by an investigator blinded to the treatment groups as previously described [14].

### Hematoxylin and eosin (HE) staining

Rats were euthanized at 72 h after the MCAO operation for HE staining. Left brains were quickly removed and further fixed using 4% paraformaldehyde overnight. Then brain tissues were equilibrated in a phosphate-buffered 30% sucrose solution, embedded in paraffin, and cut into 6-μm coronal sections.

Three rats of each group were assigned to HE staining. Sections of brain tissues embedded in paraffin were stained with HE for routine histological examinations and the morphological changes were observed under a light microscope (IX81; Olympus, Tokyo, Japan).

### Fluoro-jade B staining

Fluoro-Jade B is a high affinity fluorescent marker for the localization of neuronal degeneration. Fluoro-Jade B staining was performed using a modification of a previously published procedure [15]. Briefly, the sections were immersed in a solution containing 1% sodium hydroxide in 80% alcohol for 5 min, followed by 2 min in 70% alcohol and 2 min in distilled water. The slides were then transferred to a solution containing 0.06% potassium permanganate for 10 min, then incubated in the staining solution of Fluoro-Jade B (Millipore, Chemicon, USA) for 20 min. After washing 3 times in distilled water, the sections were allowed to dry, immersed in xylene, coverslipped, and examined using a fluorescence microscope with 450–490 nm excitation light.

### Immunofluorescence analysis

Immunofluorescence analysis of the paraffin embedded tissues was performed using standard protocols provided by the antibody manufacturers. Briefly, sections were fixed in 4% paraformaldehyde, blocked with 10% normal goat serum, and then incubated overnight at 4 °C with the primary antibodies: rabbit anti-TNF-α (Abcam, Cambridge, MA; 1:200), mouse anti-Iba1 (Abcam; 1:100), mouse anti-IL-6 (Abcam; 1:200,), mouse anti-OX-42 (Abcam; 1:100), rabbit anti-pSTAT3 (Cell Signaling Technology, Danvers, MA, USA; 1:100). Thereafter, sections were briefly washed with PBS and incubated for 1 h at room temperature with appropriate secondary antibodies (tetramethylrhodamine -conjugated anti-mouse IgG; fluorescein isothiocyanate -labeled anti-rabbit IgG; Abcam; 1:100). 4′, 6-diamidino-2- phenylinedole (DAPI; Solarbio, Beijing, China; 1 μg/ml) was used to dye the nuclei before 10 min of mounting. The sections were then analyzed with an Olympus IX81 microscope (Olympus, Tokyo, Japan) and Image-Pro Plus 6.0 software.

### Western Blot assay

The protein levels of pSTAT3, two microglia markers Iba1and OX-42, and proinflammatory cytokines TNF-α and IL-6 in the ipsilateral ischemic hemisphere (including brain cortex and hippocampus) were measured by Western blot. The protein concentration in the supernatant was measured with the bicinchoninic acid (BCA) protein assay Kit (Pierce, Rockford, IL, USA). Equivalent amounts of protein were separated on 10% sodium dodecyl sulfate-polyacrylamide gel electrophoresis. The electrophoretic transfer of proteins from polyacrylamide gels to nitrocellulose blotting membranes (Millipore, Bedford, MA, USA) was performed according to the wet electrical transfer method. The membranes were blocked with 5% non- fat dry milk in tris-buffered saline and tween 20 (TBST) and subsequently incubated with mouse anti-STAT3 antibody (Cell Signaling Technology; 1:1000), rabbit anti-phosphorylated STAT3 at Tyr705 (1:1000, Cell Signaling Technology), Mouse anti- Iba-1 (Abcam; 1:1000), mouse anti- OX-42 (Abcam; 1:1000), and mouse anti-β-actin (Cell Signaling Technology; 1:2000). After washing in TBST, immunoblots were incubated with horseradish peroxidase-conjugated secondary antibodies (HRP-linked anti-rabbit IgG; HRP-linked anti-mouse IgG; Cell Signaling Technology; 1:2000) for 1 h at room temperature. The immunoblots were developed with chemiluminescence reagents (Millipore, Bedford, MA), and then observed using the ChemiDoc™ XRS+ Imaging System (Bio-Rad, Hercules, California, USA). The densities of the immunobands were quantitated by Image J 1.4.3.67.

### Statistical analysis

Data were expressed as mean ± S.D. Variance was analyzed by a one-way ANOVA followed by Student-Newman-Keuls test. Statistical analysis was performed with SPSS v.16.0. Values of *p* < 0.05 were considered statistically significant.

## Results

### Effects of Hcy on brain infarction and neural cell injury

**Fig. 1:**
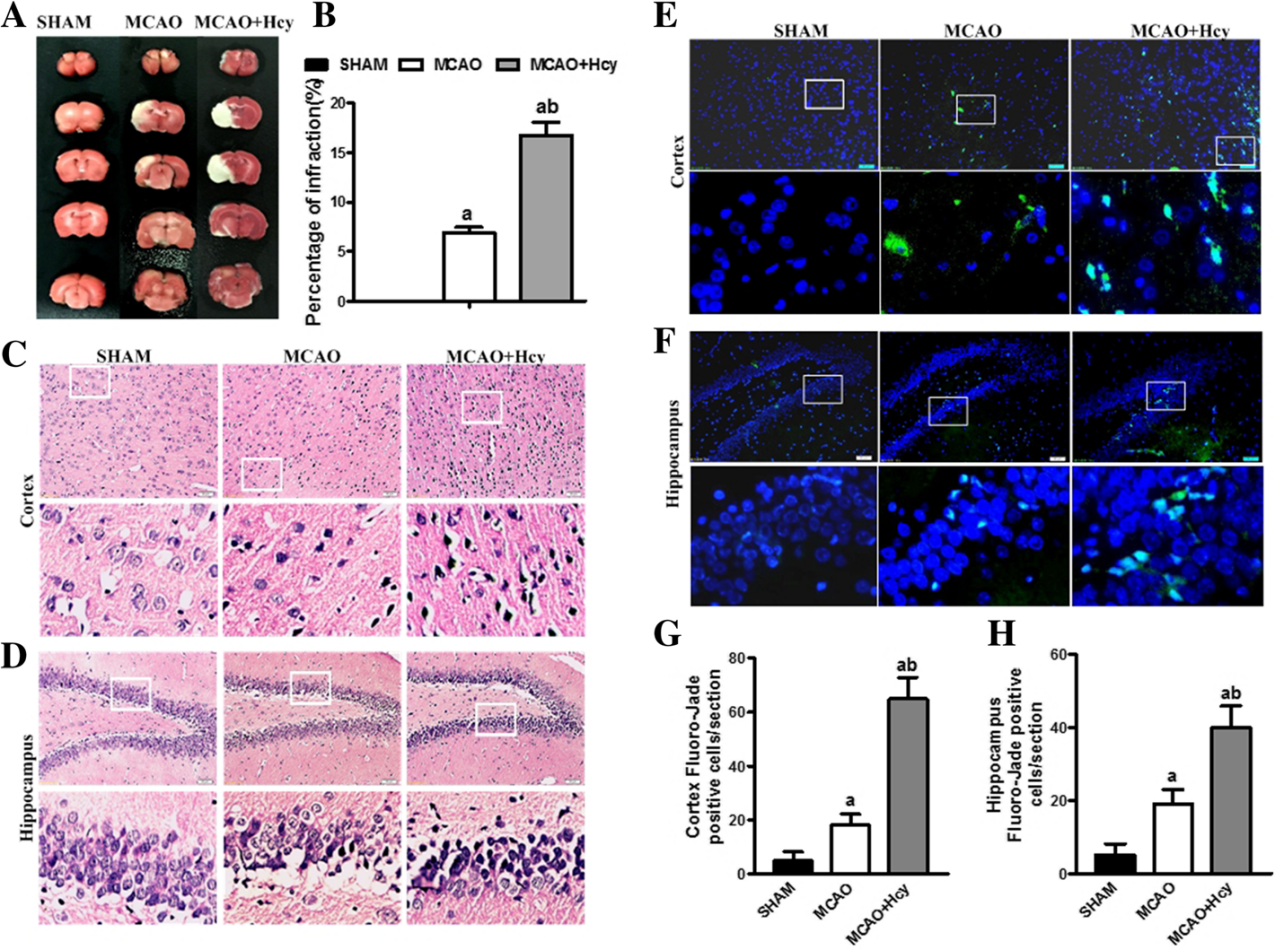
Effect of homocysteine on cerebral infarct volume and neural cell morphological structure in the rats at 72 h post-MCAO. **a** Representative photographs of TTC-stained brain coronal sections showing the increase in infarct (white) by Hcy treatment. **b** Bar graph showing the infarct volume expressed as the percentage of the whole brain volume. Treatment with Hcy greatly increased the infarct volume compared with the MCAO group. The data are expressed as the means ± SD (*n* = 3 each group). ^a^ *p* < 0.05 vs. the SHAM group, ^b^ *p* < 0.05 vs. the MCAO group. **c**, **d** Cell histopathological changes in the cerebral cortical and DG region of the hippocampus by HE staining. Bar = 50 μm. **e**, **f** Representative microphotographs by fluorescent microscopy show Fluoro Jade B+ (green)-labeled neurodegeneration in the brain cortex and hippocampus. Quantitative analysis of Fluoro-Jade B+ positive cells in the brain cortex (**g**) and hippocampus (**h**) was shown. Data are presented as the mean ± SD for 6 animals in each group (8–10 histological sections per brain). Bar = 50 μm. DAPI (blue) a nuclear marker

First, we explored the possible neurotoxic effects of Hcy in the MCAO rat model. TTC-stained brain sections were evaluated 24 h after MCAO and reperfusion. As shown in Fig. 1, no infarct area was found in the sham-operated group, because the rats in this group underwent a similar surgical procedure but were not subjected to MCAO. In the MCAO rats; however, the unstained white infarct zone was visible in the ipsilateral ischemic hemisphere, with an infarct volume of 6.9 ± 0.5% of the entire brain volume. Hcy treatment markedly enlarged the infarct volume to 16.8 ± 1.3%, compared with the MCAO group (*p <* 0.05; Fig. 1a, b).

Neuronal morphology of the brain cortex and the DG region of the hippocampus after cerebral ischemia/reperfusion was observed by HE staining72 h after reperfusion. In the SHAM group, most neurons were arranged in a regular pattern and contained the large and round nuclei. In the MCAO group, neurons were disarranged, many nuclei have become pyknotic (shrunken and dark), and they were surrounded by swollen cellular processes in the brain cortex and the DG region of the hippocampus after cerebral ischemia/reperfusion. Compared with the MCAO group, the pathological changes of the MCAO + Hcy group were magnified (Fig. 1 c, d).

Next, we tested neuronal degeneration by the Fluoro-Jade B assay 24 h post-ischemia. Few Fluoro-Jade B-labeled cells were detected in ipsilateral SHAM-controlled animals. However, in MCAO group, the number of the positive cells was significantly increased, compared to SHAM group. Furthermore, the fluorescence intensity of Fluoro-Jade B staining was notably higher in cerebral cortex and hippocampus in MCAO + Hcy group compared to the MCAO group (*p* < 0.05; Fig. 1 e–h).

In addition, Fluoro-Jade B staining was also used to detect whether Hcy or AG490 themselves caused cell injury in control animals. The results showed that few and sparse positive cells could be found in cerebral cortex and hippocampus in SHAM, SHAM + Hcy and SHAM + Hcy + AG490 groups. No differences were observed among the three experimental groups (data not shown). Thus, it is evident that Hcy or AG490 themselves did not significantly affect the uninjured brains. This suggested that Hcy may enhance the vulnerability of neuronal cells to the ischemic injury.

### Hcy raised MCAO-induced microglial activation

**Fig. 2:**
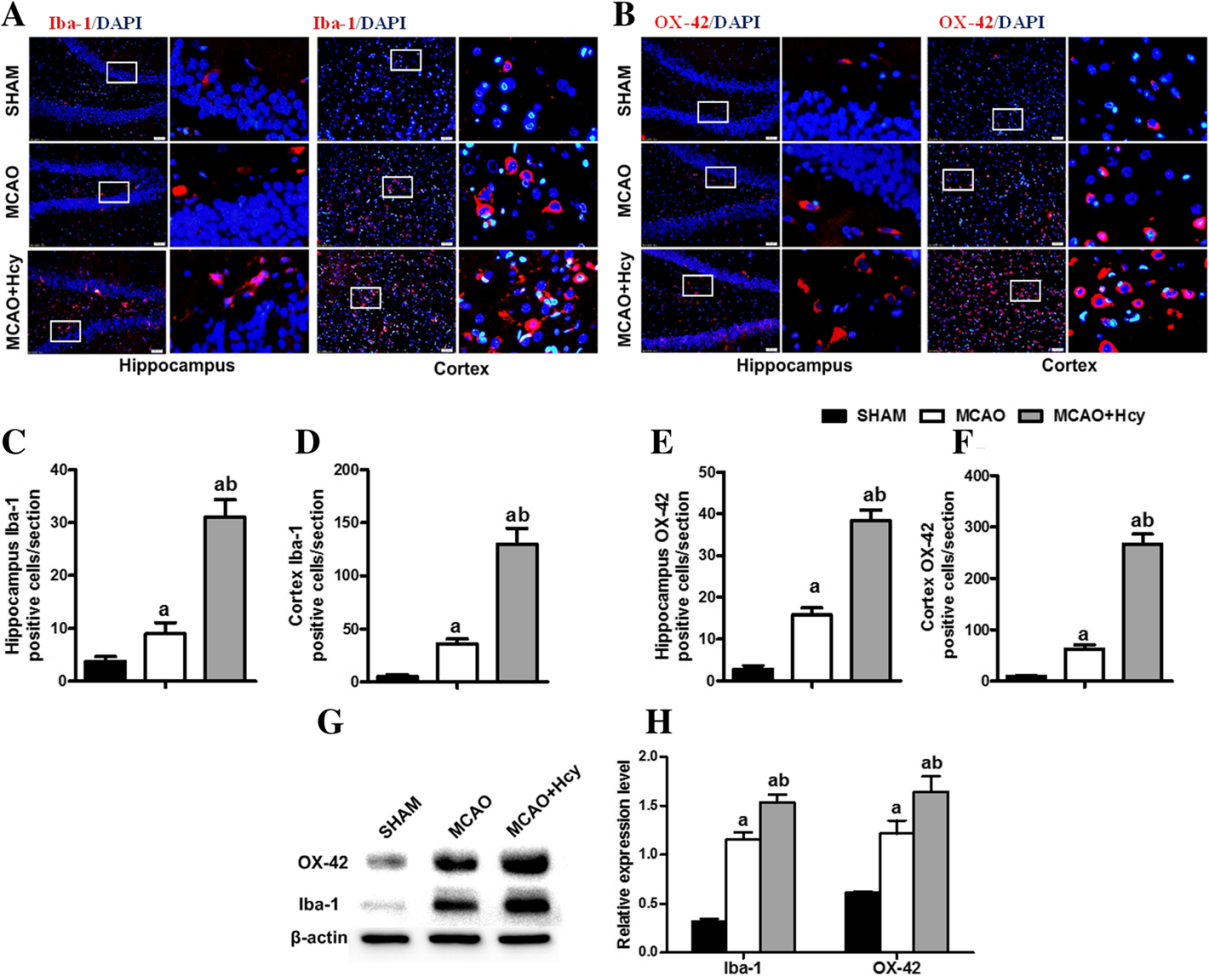
Hcy raised the MCAO-induced expression of Iba1 and OX-42 in the ipsilateral ischemic hemisphere. **a**, **b** Immunofluorescence analysis of Iba1 and OX-42 (two microglia markers) 24 h following MCAO injury. Representative images of Iba1 (red) and OX-42 (red) staining in DG area of the hippocampus and the cerebral ischemic cortex. Nuclei are stained with 4, 6-diaminido-2-phenylindole (DAPI, blue). Overlays demonstrate the colocalization of Iba1 (or OX-42) and DAPI. Each right-hand column depicts a magnified image of the rectangular region of the corresponding image in the left column. Bar = 50 μm. **c**, **d** Quantification of Iba1-positive cells in the post-ischemic hippocampus and cerebral cortex. **e**, **f** Quantification of OX-42-positive cells in the post-ischemic hippocampus and cerebral cortex. The data are presented as the mean ± SD. Six rats in each group and 10 fields for each rat were examined. **g** The expression of Iba1or OX-42 was detected by Western blot. Representative Western blot showing the activation of microglia in the ipsilateral ischemic hemisphere extracts using an Iba-1(or OX-42) antibody. **h** The levels of Iba1 and OX-42 proteins were quantified and normalized to ß-actin levels. The data are presented as the mean ± SD (*n* = 5 each group). ^a^ *p* < 0.05 vs. the SHAM group, ^b^ *p* < 0.05 vs. the MCAO group

To determine whether cortical and hippocampal microglia are capable of responding to Hcy, immunostaining for Iba1 and OX-42, two microglia specific markers, was performed to assess the pathological changes of microglia in the ipsilateral hemisphere. Figure 2 shows that there were only a few Iba1 (OX-42)-immunoreactive cells in the cerebral cortex and hippocampus in the sham-operated group. The strikingly increased number of Iba1 (OX-42)-positive cells was observed in the cortical ischemic penumbra and hippocampus, which were further raised by Hcy treatment compared with the MCAO group (*p* < 0.05; Fig. 2a–f).

These results were also confirmed by western blot analysis. We assessed Iba1 (OX-42) protein expression of the ipsilateral ischemic hemisphere extracts by Western Blot. The results showed that Iba1 (OX-42) protein expression increased after Hcy treatment for 24 h, compared with MCAO group (*p* < 0.05; Fig. 2g, h).

**Fig. 3:**
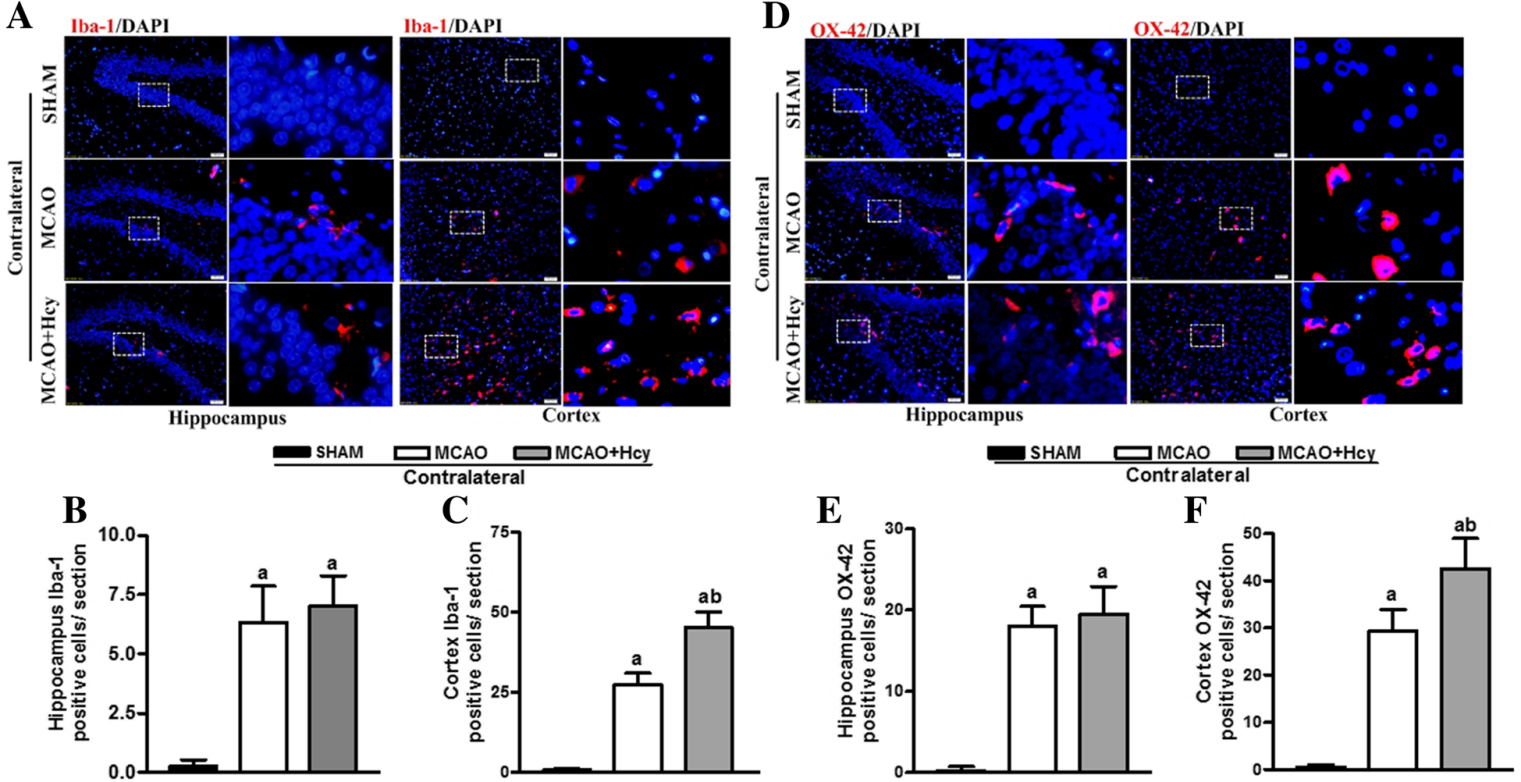
The effect of Hcy on the expression of Iba1 and OX-42 in the contralateral nonischemic side. **a**, **d** Representative images of Iba1 (red) and OX-42 (red) staining in DG area of the hippocampus and the cerebral ischemic cortex. The blue staining represents DAPI. Overlays demonstrate the colocalization of Iba1 (or OX-42) and DAPI. Each right-hand column depicts a magnified image of the rectangular region of the corresponding image in the left column. Bar = 50 μm. **b**, **c** Quantification of Iba1-positive cells in the post-ischemic hippocampus and cerebral cortex. **e**, ﻿**f** Quantification of OX-42-positive cells in the post-ischemic hippocampus and cerebral cortex.The data are presented as the mean ± SD. Five rats in each group and 10 fields for each rat were examined. ^a^ *p* < 0.05 vs. the SHAM group, ^b^ *p* < 0.05 vs. the MCAO group

Next, we examine the effect of Hcy on the microglial changes in the contralateral non-ischemic side. As shown in Fig. 3, a statistically significant increase in the number of Iba1 or OX-42 positive cells was detected in the contralateral hippocampus and cortical region in MCAO group, compared to SHAM group (*p* < 0.05). In MCAO + Hcy group, a slight increase, but not significant, in the number of positive cells was visible within the contralateral hippocampus (*p* > 0.05). In contrast, the number of Iba1 or OX-42 positive cells in the contralateral brain cortex was more significantly increased in Hcy-treated group (*p* < 0.05), compared to MCAO group, although this increase was not significant in the hippocampus.

### Hcy exposure induced IL-6 and TNF-α cytokine secretion in ischemic brains

**Fig. 4:**
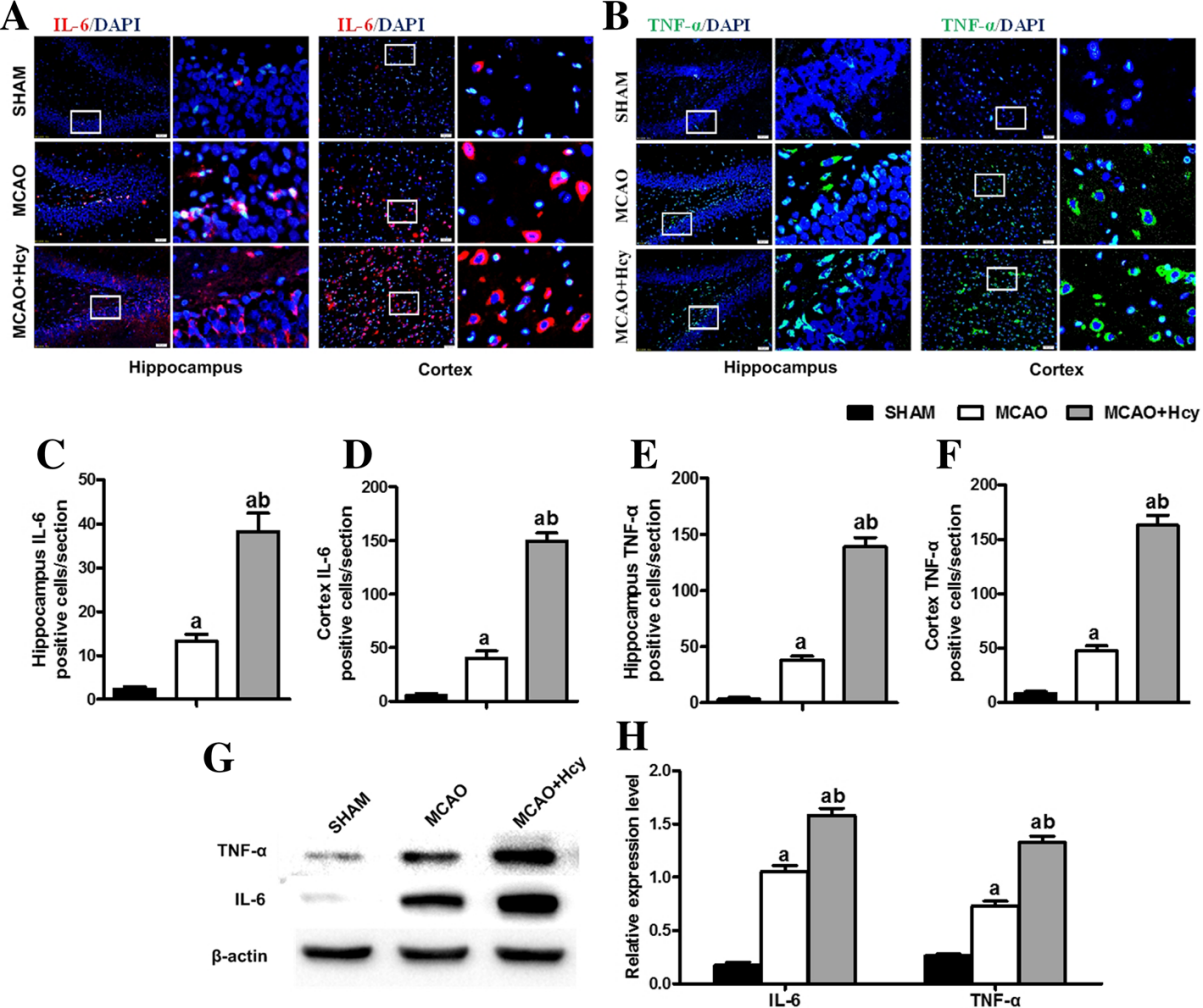
Hcy induced the secretion of proinflammatory cytokines TNF-α and IL-6 in ischemic brain injury. **a, b** Representative photomicrographs of the immunofluorescence staining of IL-6 (red) and TNF-α (green) in DG area of the hippocampus and the cerebral ischemic cortex. Nuclei are stained blue with DAPI. Each right-hand column depicts a magnified image of the rectangular region of the corresponding image in the left column. **c**-**f** Bar graphs show semi-quantification of fluorescence intensity for TNF-α and IL-6. Values are mean ± SD. Six rats in each group and 10 fields for each rat were examined. Bar = 50 μ m. **g, h** Western Blot analysis of TNF-α and IL-6. β-actin protein was used here as an internal control. The data are presented as the mean ± SD for five independent experiments. ^a^ *p* < 0.05 vs. the SHAM group, ^b^ *p* < 0.05 vs. the MCAO group

Proinflammatory mediators (such as IL-6 family of cytokines and TNF-α) have been implicated in a variety of neurotoxic conditions, often in association with the activation of microglia [16–18]. To assess the effect of Hcy on the neuroinflammatory response induced by MCAO, immunostaining was used to detect the levels of TNF-α and IL-6 production in DG area of the hippocampus and the cerebral ischemic cortex. As shown in Fig. 4a–f, the proinflammatory cytokines TNF-α and IL-6 was not significantly detected in the sham-operated group, whereas their immunoreactivities strongly increased in the cerebral ischemic cortex and hippocampus compared with the sham-operated group (*p* < 0.05). After treatment with Hcy, TNF-α and IL-6 secretion was further elevated, compared with the MCAO group (*p* < 0.05).

Coincident with the change in Iba1-positive (or OX-42) cells in the cerebral ischemic cortex and the hippocampus, Western Blot analysis also showed that the protein expressions of TNF-α and IL-6 were significantly augmented in the ipsilateral ischemic hemisphere extracts after Hcy treatment (*p* < 0.05, Fig. 4g, h). These results suggest that Hcy can upregulate MCAO-induced neuroinflammation.

### Phosphorylation of STAT3 in microglia was upregulated by Hcy exposure

**Fig. 5:**
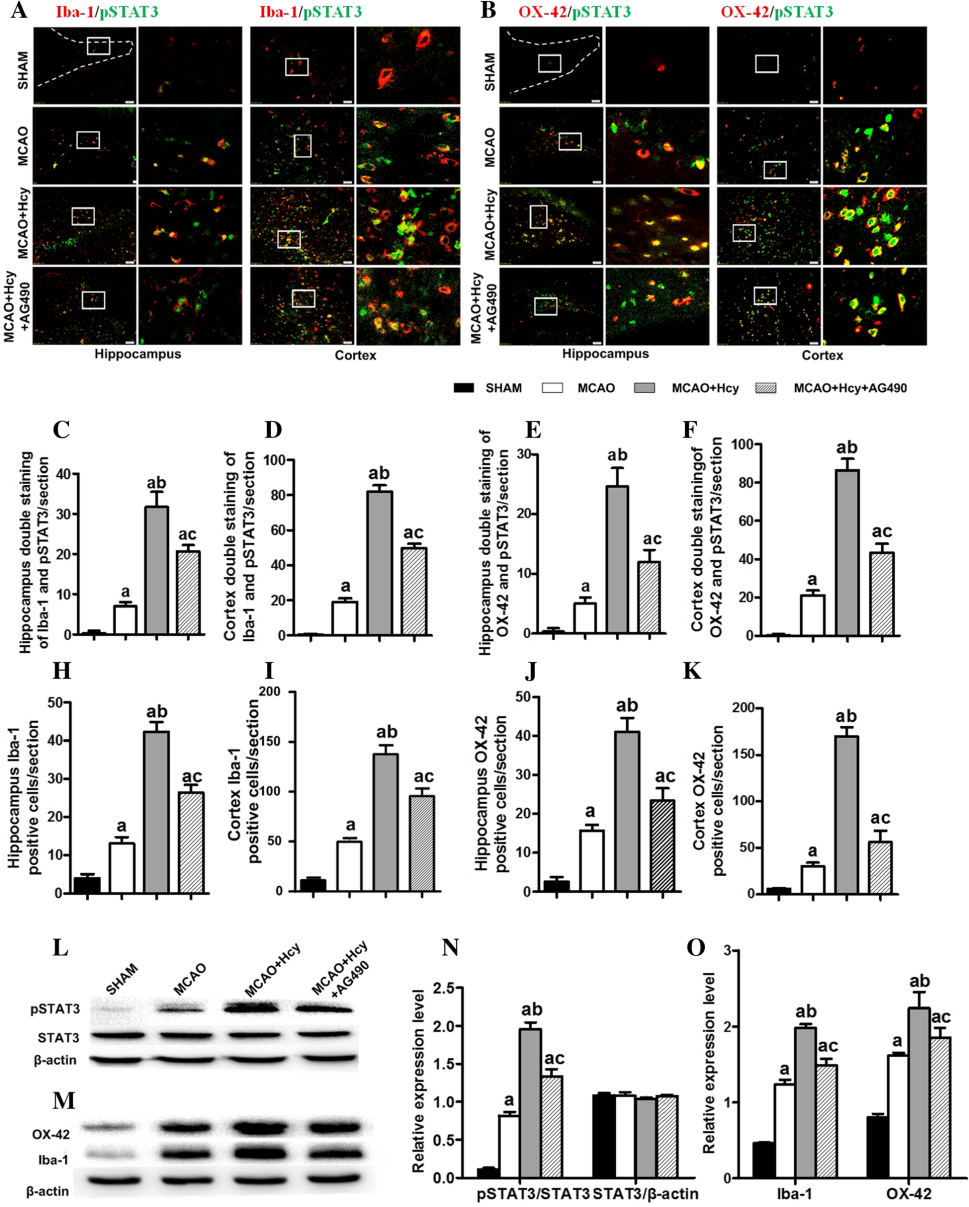
Hcy activates microglia by regulating STAT3 signaling pathway which occurs in microglia following stroke. **a, b** Representative photomicrographs of double immunofluorescence staining of pSTAT3 (green) with Iba-1 (red) or OX-42 (red), the markers of activated microglia, in the DG region of the rat hippocampus and the cerebral ischemic cortex. Overlays demonstrate the colocalization of pSTAT3 and Iba-1 (or OX-42). Each right-hand column depicts a magnified image of the rectangular region of the corresponding image in the left column. Scale bars =50 μm. **c**-**f** Quantitative assessment of pSTAT3/Iba-1 (or OX-42) double positive cells in the rat brain hippocampus and the cortex (*n* = 6, 10 fields/brain). **h-k** Quantitative assessment of Iba-﻿1 (or OX-42) positive cells in the rat bran hippocampus and cortex ﻿(﻿n=6, 10﻿ fields/b﻿﻿r﻿ain﻿). **l** The phosphorylated and total protein expression of STAT3 was detected by Western Blot assay. **m** Representative western blot for Iba-1 (or OX-42). **n** Bar graphs show semiquantitative levels of pSTAT3/STAT3 and STAT3/ß-action as determined by band density analysis. **o** Semiquantitative analysis of Iba-1/ß-action and OX-42/ß-action, respectively. The data are presented as means ± SD from three independent experiments. ^a^ *p* < 0.05 vs. the SHAM group, ^b^ *p* < 0.05 vs. the MCAO group, ^c^ *p* < 0.05 vs. the MCAO + HCY group

The STAT3 signaling pathway has been suggested to play a critical role in neuroinflammation and the activation of microglia following neurotoxic insults and stroke. To investigate whether the STAT3 signal is involved in Hcy-induced the activation of microglia, we co-stained for pSTAT3 and for two microglia markers: Iba1 and OX-42. Double immunofluorescence demonstrates that pSTAT3-labeled cells were colocalized with Iba-1 or OX-42 antigen. The immunoreactivity of pSTAT3 in microglia was not significantly detected in the sham-operated group, which was strongly increased both in the brain cortex and the DG region of the hippocampus in MCAO group (Fig. 5a, b). Injections of Hcy further significantly increased the number of pSTAT3 and Iba1 (OX-42) double positive cells in the hippocampus (Fig. 5 c, e, *p* < 0.05) and the cerebral ischemic cortex (Fig. 5d, f, *p* < 0.05). Furthermore, the Western Blot results revealed the increased level of pSTAT3 in brain tissue extracts in MCAO group compared with the sham-operated group. Hcy treatment further increased the phosphorylation of STAT3 compared with the MCAO group (*p* < 0.05), despite the fact that the total levels of STAT3 were not altered in microglia (Fig. 5l, n).

The Janus family of tyrosine kinase 2 (JAK2), an upstream kinase, mediates STAT3 phosphorylation at Tyr-705 site. Numerous studies have shown that specific inhibition of STAT3 activation by JAK2-specific inhibitor AG490 blocked STAT3 phosphorylation [19, 20]. Both Western Blot and immunofluorescence analysis revealed that AG490 treatment abolished the increase in Hcy-induced phosphorylation of STAT3 (Fig. 5).

### JAK2 inhibitor, AG490, partially attenuated microglia activation induced by Hcy treatment

To further assess the potential role of the STAT3 pathway in Hcy-induced activation of microglia, we next examined whether a specific JAK2 inhibitor AG490 could affect Hcy-induced increase of Iba1 and OX-42 protein expression in the cerebral cortex and the hippocampus. The immunofluorescence analysis showed that the number of Iba1 (OX-42)-positive cells raised by Hcy was significantly decreased by AG490 administration compared with the MCAO group (*p* < 0.05, Fig. 5h–k). Similarly, Western Blot showed that AG490 partially blocked the expression of Iba1 (OX-42) in brain tissue extracts after Hcy treatment, compared with the MCAO group (*p* < 0.05, Fig. 5m, o). It suggested that Hcy-induced microglial activation may be mediated, at least in part, by JAK2-STAT3 signaling pathway.

### AG490 partially attenuated Hcy-induced TNF-α and IL-6 production

**Fig. 6:**
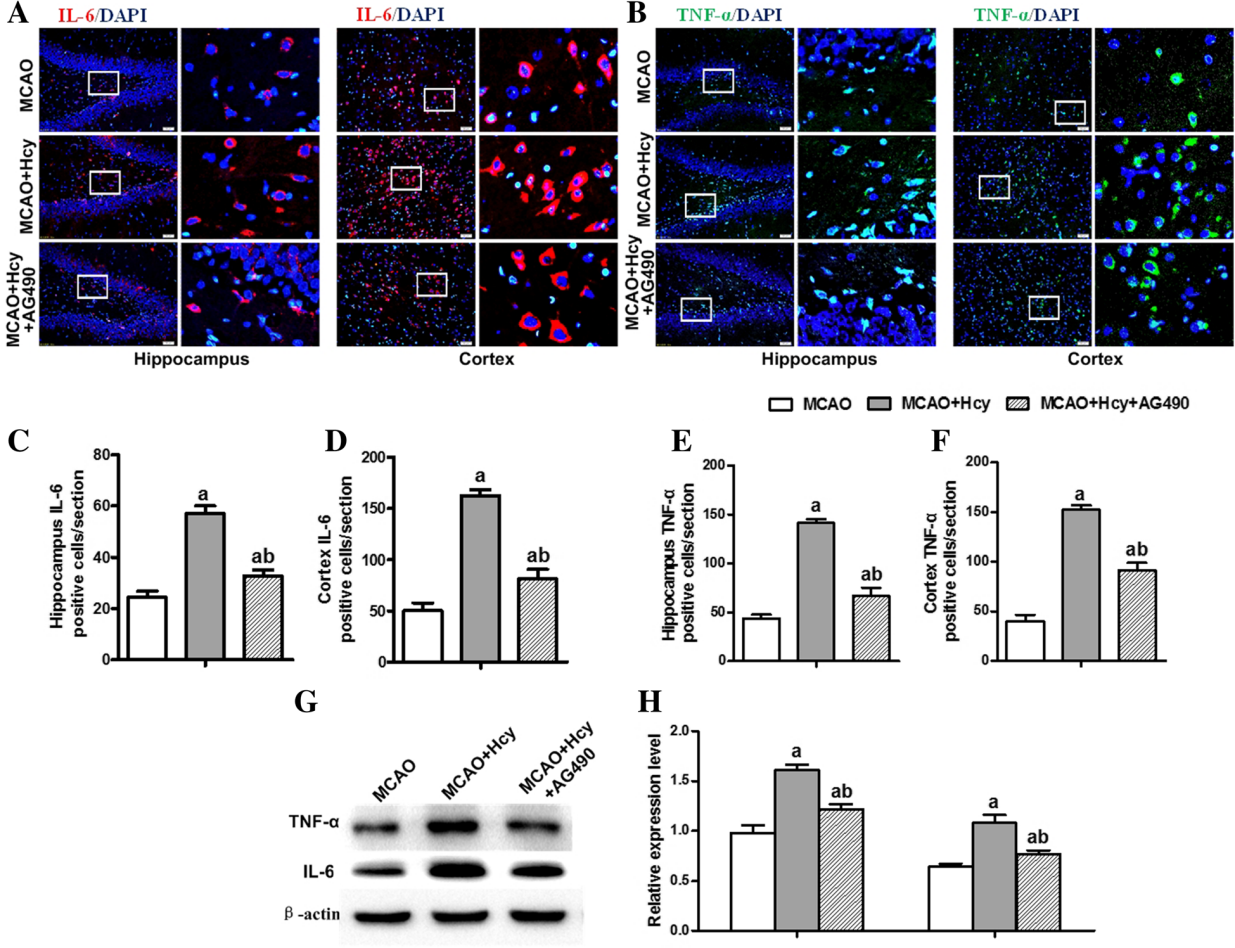
The effects of AG490 on TNF-α and IL-6 production induced by Hcy. **a, b** TNF-α (green) and IL-6 (red﻿) immunoreactivity in the DG region of the rat hippocampus and the cerebral ischemic cortex after Hcy and AG490 treatment. The blue staining represents DAPI. Scale bar = 50 μm. **c**-**f** Graph showing the mean fluorescence intensity for TNF-α and IL-6 (*n* = 6, 10 fields/brain). **g** Representative bands of TNF-α and IL-6 by western blot analysis. β-actin protein was used here as an internal control. **h** Bar graphs show semiquantitative levels of TNF-α and IL-6 as determined by band density analysis. The data are presented as the mean ± SD from four independent experiments. ^a^ *p* < 0.05 vs. the SHAM group, ^b^ *p* < 0.05 vs. the MCAO group

The functional association between cytokine signaling and JAK-STAT signaling prompted us to examine the involvement of STAT3 in Hcy-induced inflammatory responses. We explored the effects of AG490 on Hcy-induced release of cytokines TNF-α and IL-6 by immunofluorescence staining and Western Blot analysis. Immunohistochemical analysis revealed that the decrease in the number of TNF-α-positive and IL-6-positive cells was observed in the hippocampus DG region and the cerebral ischemic cortex of the rats receiving AG490 and Hcy combined treatment compared with those of MCAO rats treated with Hcy alone (Fig. 6a–f).

Consistent with immunofluorescence staining results, Western Blot analysis showed that the expression of TNF-α and IL-6 in Hcy-injected rats was significantly higher than the expression in the ischemic brains but these elevations were remarkably inhibited by the AG490 treatment (Fig. 6g, h). Thus, the above results demonstrated that AG490 partially abolished the increase in Hcy-induced TNF-α and IL-6 production.

## Discussion

The ischemic stroke is one of the common causes of morbidity and mortality worldwide. There are many causes of ischemic stroke, and hyperhomocysteinemia is one of the documented causes. Hyperhomocysteinemia originates from a deviation in the methionine-homocysteine metabolism including disturbances of enzymes, vitamin deficiencies, and different other factors [21, 22]. Although it has been shown that Hcy has a neurotoxic effect on ischemic brains, its roles in modulating microglial activation and inducing inflammation have not been documented. The present study reveals that Hcy treatment enhanced brain injury, induced activation of microglia, and triggered the expression of pro-inflammatory cytokines in the brain cortex and the DG region of the hippocampus using a rat MCAO model. Moreover, to our best knowledge, this is the first study to provide evidence that changes of STAT3 activities located in microglia involve Hcy-induced microglia activation and inflammation responses following stroke.

The previous studies demonstrated that the main cell types of the central nervous system involve Hcy neurotoxicity. High level of Hcy can cause the toxic effect, leading to neuron and neural cell death [23, 24] while low dose can promote glial cell proliferation [25]. In addition, some studies suggest that the neurotoxicity of Hcy may involve negative regulation of neural stem cell (NSC) proliferation [26]. Several reports have also found that Hcy promotes proliferation and activation of microglia through induction of NAD(P)H oxidase in vitro [25, 27]. Low-dose Hcy not only induced Bv2 cell proliferation but also activated these cells in a dose- and time-dependent manner. In our study, Hcy induced microglia activation in ischemic brain. This result is in line with in vitro study. Meanwhile, since Hcy affected the activities of several main brain cells, complex pathogenetic mechanisms may be involved in brain injury caused by Hcy after cerebral ischemia.

Microglia represent 5–20% of the total glial population and are key modulators of the immune response in the brain [28, 29]. Similar to the role of peripheral macrophages, Microglia are now known as the first line of immune defense against central nervous system (CNS) injuries and disorders. These highly plastic cells play dualistic roles in neuronal injury and recovery. Some studies have found that under physiologic conditions, resident microglia are quiescent and scattered throughout the CNS [30]. Occasionally microglia are moderately activated to play a classic role as “scavenger” for the maintenance and restoration of the CNS [31]. The moderate activation of microglia contributes to nervous system repair by engulfing pathogens within brain tissue and releasing neurotrophic factors. However, excessive and prolonged activation of microglial cells can lead to a variety of pathological damages in the central nervous system [3, 32]. Vehmas et al. [33] have confirmed that the amount of activated microglial cells is positively correlated with the progress of Alzheimer’s disease. In our models, Hcy treatment activated microglial cells, markedly enlarged the infarct volume and induced cell injury, so the hyperactivation of microglial cells caused by Hcy appeared to be harmful for the ischemic brain. Thus, whether the activation of microglial cells is beneficial or detrimental appears to depend on the extent of cell activation.

In most cases, activated microglia initiate neuroinflammation by producing cytotoxic and inflammatory factors such as the cytokines IL-1β, TNF-α and IL-6, thereby aggravating brain damage [34]. High concentration of TNF-α has direct toxic effect on neurons and neural cells. TNF-α transgenic mice have severe inflammation and exhibit brain and neurodegenerative diseases [35]. Regarding IL-6, its high expression can accelerate the pathological processes of central nervous system disorders [36]. Meanwhile, there is increasing evidence that demonstrating inflammation not only immediately affects the infarcted tissue but also causes long-term damage in the ischemic penumbra after cerebral ischemia. The present study confirms that Hcy treatment induced activation of microglia and a significant up-regulation of the inflammatory factors TNF-α and IL-6 in the cortex and hippocampus of ischemic brains. Moreover, based on results of TTC staining and morphological assays, Hcy may exert a neurotoxic effect by microglia-mediated neuroinflammation injury.

To explore the potential mechanisms of the microglia-inflammatory enhancing effect of Hcy, we focused on investigating JAK2/STAT3 pathway. JAK2/STAT3, as critical immunological signaling molecules, are normally expressed in the brain and play a vital role in regulating microglial activation and inflammatory response [37, 38]. STAT3, an important downstream regulatory molecule of the JAK/STAT pathway, is a well-established regulator of inflammatory gene expression and a marker of CNS damage [39]. Expression levels of STAT3 have been shown to be enhanced in reactive microglia cells when the brain suffers focal ischemic injury [40] Aberrantly activated STAT3 has also been reported to involved in the neuroinflammatory injury after ischemic stroke. To investigate the potential function of the JAK2-STAT3 pathway in Hcy-induced microglia activation and inflammatory response following MCAO, we examine whether the JAK2 inhibitor AG490 could affect Hcy-induced pSTAT3, the microglia specific markers Iba-1and OX-42, and the proinflammatory mediators TNF-α and IL-6 expression. Our results revealed that the activation of STAT3 in microglia and secretion of TNF-α and IL-6 were significantly upregulated in Hcy-treated ischemic brains and this effect was reversed by AG490. These data provide further evidence that that the STAT3 expression raised by Hcy treatment might be involved in microglial activation and the neuroinflammatory injury in MCAO rats.

Focal ischemia-induced STAT3 phosphorylation was previously reported to be localized in various cell types including microglia/macrophages, astrocytes and neurons [39, 41, 42]. Activated microglia/macrophages (ED1 and OX42/Cd11b positive, microglia/macrophage markers) were observed to be the predominant cell type that showed pSTAT3 immunostaining at 24 and 72 h after brain ischemia/reperfusion injury [12]. Moreover, it has been reported that pSTAT3 immunoreactivity was also present in different brain regions following stroke. For instance, Planas et al. [39] showed that a transient episode of MCAO induced a strong microglial response. This was accompanied by increased expression of STAT3 in the ipsilateral cortex and striatum. However, little is known about changes in activation of microglial STAT3 in rat hippocampus after brain ischemia, despite the finding that hippocampus is one of the most vulnerable brain regions to ischemic damages. The present study demonstrated that a strong induction of microglial STAT3 occurred in rat hippocampus after cerebral ischemia by immunohistochemistry. In future study, two points will still need to be clarified: whether the activation of STAT3 in neurons, reactive astrocytes and microglia would play different roles in ischemia-induced injury and whether STAT3 activation in different brain regions may serve different functions.

## Conclusions

Hcy further enhanced MCAO-induced microglial activation and inflammatory cytokine secretion including TNF-α and IL-6 production. AG490, a JAK inhibitor, diminished Hcy-enhanced phosphorylation of STAT3, significantly reduced Hcy-induced microglia cell activation and TNF-α and IL-6 production, indicating that Hcy triggered brain injury possibly through the JAK2/STAT3 signaling pathway. Our data provide significant new information regarding the molecular mechanisms underlying Hcy-induced microglial inflammation, and such knowledge will assist in the better understanding of the pathogenesis of brain disease.

## Additional file

Additional file 1: Video S1. Rat tail vein injection. Homocysteine was injected into the tail vein of a rat. (MP4 8751 kb)

## Abbreviations

AG490: α-cyano-(3,4-dihydroxy)- N-benzylcin-namide
BCA: Bicinchoninic acid
CNS: Central nervous system
DAPI: 4,6-diaminido-2-phenylindole
DG: Dentate gyrus
Hcy: Homocysteine
HE: Hematoxylin-eosin staining
IL-6: Interleukin 6
JAK2: The Janus family of tyrosine kinase 2
MCAO: Middle cerebral artery occlusion reperfusion group
ROS: Reactive oxygen species
SHAM: Sham operation control group
STAT3: Signal transducer and activator of transcription 3
TBST: Tris buffered saline with Tween 20
TNF-α: Tumor necrosis factor α
TTC: 2,3,5-triphenyltetrazolium chloride
